# Population pharmacokinetics of artesunate and dihydroartemisinin in pregnant and non-pregnant women with malaria

**DOI:** 10.1186/1475-2875-10-114

**Published:** 2011-05-08

**Authors:** Carrie A Morris, Marie A Onyamboko, Edmund Capparelli, Matthew A Koch, Joseph Atibu, Vicky Lokomba, Macaya Douoguih, Jennifer Hemingway-Foday, David Wesche, Robert W Ryder, Carl Bose, Linda Wright, Antoinette K Tshefu, Steven Meshnick, Lawrence Fleckenstein

**Affiliations:** 1 University of Iowa, College of Pharmacy, 115 South Grand Avenue, Iowa City, IA 52242, USA; 2 Kinshasa School of Public Health, Kinshasa, The Democratic Republic of Congo; 3 University of California, San Diego, CA, USA; 4 RTI International, Research Triangle Park, NC, USA; 5 Aeras Global TB Vaccine Foundation, Rockville, MD, USA; 6 David Wesche Consulting LLC, Ann Arbor, MI, USA; 7 University of North Carolina at Chapel Hill, NC, USA; 8 National Institute of Child Health and Human Development, NIH, Rockville, MD, USA; 9 UNC Gillings School of Global Public Health Department of Epidemiology, Chapel Hill NC, USA

## Abstract

**Background:**

The World Health Organization endorses the use of artemisinin-based combination therapy for treatment of acute uncomplicated falciparum malaria in the second and third trimesters of pregnancy. However, the effects of pregnancy on the pharmacokinetics of artemisinin derivatives, such as artesunate (AS), are poorly understood. In this analysis, the population pharmacokinetics of oral AS, and its active metabolite dihydroartemisinin (DHA), were studied in pregnant and non-pregnant women at the Kingasani Maternity Clinic in the DRC.

**Methods:**

Data were obtained from 26 pregnant women in the second (22 - 26 weeks) or the third (32 - 36 weeks) trimester of pregnancy and from 25 non-pregnant female controls. All subjects received 200 mg AS. Plasma AS and DHA were measured using a validated LC-MS method. Estimates for pharmacokinetic and variability parameters were obtained through nonlinear mixed effects modelling.

**Results:**

A simultaneous parent-metabolite model was developed consisting of mixed zero-order, lagged first-order absorption of AS, a one-compartment model for AS, and a one-compartment model for DHA. Complete conversion of AS to DHA was assumed. The model displayed satisfactory goodness-of-fit, stability, and predictive ability. Apparent clearance (CL/F) and volume of distribution (V/F) estimates, with 95% bootstrap confidence intervals, were as follows: 195 L (139-285 L) for AS V/F, 895 L/h (788-1045 L/h) for AS CL/F, 91.4 L (78.5-109 L) for DHA V/F, and 64.0 L/h (55.1-75.2 L/h) for DHA CL/F. The effect of pregnancy on DHA CL/F was determined to be significant, with a pregnancy-associated increase in DHA CL/F of 42.3% (19.7 - 72.3%).

**Conclusions:**

In this analysis, pharmacokinetic modelling suggests that pregnant women have accelerated DHA clearance compared to non-pregnant women receiving orally administered AS. These findings, in conjunction with a previous non-compartmental analysis of the modelled data, provide further evidence that higher AS doses would be required to maintain similar DHA levels in pregnant women as achieved in non-pregnant controls.

## Background

Infection with *Plasmodium falciparum *during pregnancy can have severe health consequences for both the infected woman and her unborn child. In regions of unstable malaria transmission, in which acquired immunity to malaria is minimal, pregnant women are more vulnerable to severe falciparum malaria infection. In areas of stable malaria transmission, including most of sub-Saharan Africa, pregnant women experience higher rates of falciparum parasitaemia and severe malaria-related anaemia than their non-pregnant counterparts [1]. In these regions, placental malaria infection, characterized by *P. falciparum *sequestration in placental tissue, also represents a significant public health threat. Placental malaria, particularly when paired with maternal anaemia, can compromise foetal nutrition, resulting in intrauterine growth restriction, preterm birth, and low birth weights [1]. In sub-Saharan Africa, malaria infection in pregnancy is responsible for an estimated 20% of low birth weight deliveries and a consequent 100,000 infant deaths every year [2].

The World Health Organization (WHO) recommends artemisinin-based combination therapy (ACT) as a first-line treatment for acute, uncomplicated falciparum malaria in the second and third trimesters of pregnancy [3]. Additionally, the inclusion of artemisinin derivatives in novel intermittent preventative treatment regimens for pregnant women has been proposed as a means to combat the effects of increasing resistance to the currently used agent, sulphadoxine-pyrimethamine (SP) [4].

Despite the current and potential applications of artemisinin derivatives to the treatment and prevention of malaria in pregnant women, understanding of how the physiologic changes of pregnancy may alter the pharmacokinetics, and therefore potentially the efficacy, of artemisinin derivatives is relatively limited. To date, only three published analyses regarding the pharmacokinetics of such derivatives in pregnant women are available. Two were conducted by McGready and colleagues, who assessed the pharmacokinetics of artesunate (AS) and artemether in Thai women in the second and third trimesters of pregnancy with acute uncomplicated falciparum malaria [5,6]. Their findings suggest that in pregnant women, exposure to dihydroartemisinin (DHA), the common active metabolite of AS and artemether, is substantially lower than exposure reported in previous studies with non-pregnant adults. In a recent non-compartmental pharmacokinetic study, the clearance of DHA was determined to be faster in pregnant women than non-pregnant controls, but not than in the same women three months postpartum [7].

To date, no population pharmacokinetic analysis of an artemisinin derivative has been conducted utilizing data from not only pregnant subjects, but also non-pregnant controls. The objective of this analysis was to utilize a population pharmacokinetic approach to model the pharmacokinetics of oral AS, a water soluble artemisinin derivative, and its active metabolite DHA, in pregnant women and controls and to identify clinically relevant covariates associated with inter-individual variability in AS and DHA pharmacokinetics.

## Methods

### Study design

The clinical aspects of this single center, open label study (Clinicaltrials.gov: NCT00538382) were conducted at the Kingasani Maternity Clinic in Kinshasa, Democratic Republic of Congo (DRC). Women presenting for prenatal care at the clinic were screened for study eligibility if they were between 18 and 40 years of age and at less than 22 weeks gestation, determined by last menstrual period. Women confirmed by ultrasound to be between 8 to 21 weeks gestation (inclusive) were invited to be participants in the study. Women were asked to return between 22 to 26 weeks gestation for screening and enrollment; women not enrolled at 22 to 26 weeks were screened again at 32 to 36 weeks gestation. A cohort of non-pregnant female controls was also enrolled. At the time of enrollment, both the pregnant and non-pregnant subjects had asymptomatic *Plasmodium falciparum *parasitaemia, with a parasite density between 200 and 300,000 parasite/μL, were HIV seronegative, and were without anaemia (haematocrit >30%) or other major medical problems (e.g. chronic hypertension, diabetes, etc.). *Plasmodium falciparum *parasite density was assessed though Giemsa staining of thick and thin blood films; slide-positive infections were later PCR-confirmed using DNA extracted from dried blood spots [8]. The study protocol was approved by the ethical committees of the University of North Carolina at Chapel Hill, the Kinshasa School of Public Health, and the Research Triangle Institute. The study was carried out in accordance with the Helsinki Declaration. Only women able to understand the study protocol and who gave informed consent were enrolled in the study. Further details of the clinical and safety aspects of this trial, including additional biochemical assessments, are described by Onyamboko *et al *[7].

For pregnant subjects, pharmacokinetic studies were conducted both at the time of enrollment, as well as at three months postpartum. All subjects received 200 mg oral AS, administered as four 50 mg tablets (Guilin Pharmaceutical Co. Ltd) at the beginning of an inpatient stay at the clinic. Blood samples (5 mL) for pharmacokinetic analysis were drawn at pre-dose and at 0.25, 0.5, 0.75, 1, 1.5, 2, 3, 4, 6, and 8 hours after AS administration. Twenty-four hours following AS administration, malaria-infected women received 1725 mg SP to complete treatment. Blood sampling schedule and sample handling were uniformly applied for pregnant, postpartum, and control subjects. Blood samples were collected in pre-chilled tubes containing potassium oxalate/sodium fluoride. Following collection, tubes were placed on wet ice; within 5 minutes of collection, samples were centrifuged. Immediately following centrifugation, plasma was removed from the cells and transferred into cryovials. The plasma samples were transferred to liquid nitrogen until they could be frozen at or below -80°C in a laboratory freezer; samples were later shipped on dry ice to the Clinical Pharmacokinetics Laboratory at the College of Pharmacy, University of Iowa, where they were stored at -80°C until drug analysis was performed.

### Sample analysis

Determination of AS and DHA plasma concentrations was performed using a validated liquid chromatography-mass spectrometric method described by Naik *et al *[9] with minor modifications to allow for extraction from a smaller plasma volume. Briefly, solid phase extraction was used to extract AS, DHA, and the internal standard artemisinin from 0.25 mL of human plasma. The reconstituted extracts were chromatographed isocratically. Mass spectroscopy in positive ion mode was used to detect and quantify the compounds. The lower limit of quantification (LLQ) for both AS and DHA was 1 ng/mL. Assay validation indicated that assay precision was 5.8 - 8.6% (coefficient of variation) for AS and 6.5 - 8.2% for DHA.

### Population pharmacokinetic analysis

Nonlinear mixed effects model building was conducted using NONMEM software version 7 (ICON Development Solutions, Ellicott City, MD) [10] implemented on a Windows XP operating system (Microsoft Corporation, Seattle, WA) with a G95 Fortran compiler (Free Software Foundation, Boston, MA). Monte Carlo importance sampling expectation maximization with interaction (IMP INTER) estimation method was used to fit models. Pdx-Pop 4.0 (ICON Development Solutions, Ellicott City, MD) and Xpose version 4.1.0 (Uppsala University, Uppsala, Sweden) [11] were used in processing NONMEM 7 output. Plots were generated with TIBCO Spotfire S+ version 8.1 (TIBCO Software Inc., Palo Alto, CA) and R version 2.10.0 (Free Software Foundation, Boston, MA).

Model selection was guided by the following criteria: plausibility and precision of parameter estimates, minimum objective function value (MOFV), equal to minus twice the log likelihood function, Akaike Information Criterion, equal to MOFV plus two times the number of parameters, condition number, equal to the ratio of the largest Eigen value to the smallest Eigen value, and inspection of diagnostic plots.

Prior to modelling, AS and DHA concentrations were converted from ng/mL to nmol/L values using the compounds' respective molecular weights; the concentrations were then natural log-transformed. The 200 mg AS dose was similarly converted to the appropriate value in nmols.

Modelling was initially conducted with an aggregate data set of pregnancy, postpartum, and control observations. A structural model adequately describing data from all three groups could not be identified. As this difficulty appeared to stem from the erratic and unpredictable AS and DHA observations from the postpartum subjects, the data were subsequently divided into pregnancy/control and postpartum data sets for structural model identification. Since an adequate structural model could only be identified for the pregnancy/control data set, the *Base model development *and *Covariate model building *sections that follow describe model building using the pregnancy/control data set, with details regarding attempts to model postpartum data provided in *Results.*

### Base model development

Modelling was first performed with AS data only; first-order absorption with one-compartment and two-compartment models were fitted to these data. Alternative absorption processes were also assessed, including first-order absorption, zero-order absorption with lagged first-order absorption, first-order absorption with lagged zero-order absorption, parallel dual first-order absorption, single Weibull absorption, and transit compartment absorption. DHA data were also initially modelled independently in order to determine if a one- or two-compartment model better characterized observed concentrations.

Multiple simultaneous parent-metabolite models consisting of a one-compartment model for AS and a one-compartment model for DHA with various AS absorption types were assessed. These simultaneous models included the absorption processes assessed with AS data only, as well as parallel dual first-order absorption of both AS and DHA from a gut compartment. Complete, irreversible conversion of AS to DHA was assumed for all models [12]. Simultaneous models were implemented using ADVAN 5.

Inter-individual variability (IIV) was modelled on pharmacokinetic parameters using a log-normal distribution:

where P_i _represents the parameter estimate for individual i, P_pop _represents the population estimate for the parameter, and η_i _represents the deviation of P_i _from P_pop._.

Residual variability (RV) was modelled with an additive model for log-transformed data:

where C_ij _represents the jth observation for individual i, C_pred, ij _represents the predicted AS or DHA concentration for individual i, and ε_ij _represents the residual random error for the jth observation of individual i.

### Covariate model building

Once the optimal base model was determined, covariate analysis was undertaken to identify any covariates explaining a significant portion of the observed IIV. Covariates examined included age, weight, body mass index (BMI), baseline alpha-1-acid glycoprotein (AGP), baseline albumin, pregnancy status, and window of pregnancy. Examined covariates represented available demographic and clinical variables which could plausibly alter AS or DHA pharmacokinetics. Potential covariate-parameter relationships were identified by examining plots of covariates versus parameter estimates and covariates versus IIV. Covariate screening was also conducted using generalized additive modelling in Xpose software. Physiologically plausible covariate-parameter relationships suggested by evaluation of covariate plots and/or by generalized additive modelling were evaluated for statistical significance using a process of forward addition and backward elimination [13]. The statistical criteria for a covariate to be retained in the model during forward addition was p < 0.05; for backward elimination, the criteria was p < 0.001.

Categorical covariates were modelled using a proportional function:

where θ_1 _represents the parameter estimate in subjects with the covariate coded as 0 and θ_2 _represents the change in the parameter associated with the categorical covariate being tested.

Continuous covariates were centered on their median and modelled using a linear function:

where θ_1 _represents the parameter estimate for an individual with COV equal to COV_median_, and θ_2 _represents the change in the parameter estimate associated with the difference between COV from COV_median_.

### Model evaluation

Diagnostic plots used to assess model goodness-of-fit included observed concentrations versus population predictions, observed concentrations versus individual predictions, conditional weighted residuals (CWRES) versus population predictions, and CWRES versus time. Population predictions were obtained using the EPRED option in NONMEM 7.

One thousand bootstrap runs were conducted using Perl-Speaks-NONMEM version 3.1.0 [14] in order to assess the precision of the parameter estimates. Model stability was assessed by condition number, with a condition number less than 1000 considered indicative of model stability. The predictive ability of the model was evaluated by simulating 1000 virtual observations for each sampling time point. The observed concentrations were plotted with the 5^th^, 50^th^, and 95^th ^percentiles of the simulated data above LLQ. The percent of observed concentrations outside of the 90% prediction interval, defined by the 5^th ^and 95^th ^percentiles, were computed for both AS and DHA.

## Results

### Subject data

Demographic and clinical data for the pregnant women and controls enrolled in this study are provided in Table 1. Thirteen pregnant women were enrolled in each of the two windows of pregnancy. All pregnant (n = 26) and control subjects (n = 25) were assessed as slide positive and PCR-positive for falciparum parasitaemia at enrollment, although at the time of AS administration, typically occurring one day following enrollment, two pregnant and 11 control subjects were assessed as slide negative for parasitaemia. All infections were *P. falciparum *monoinfections with the exception of one pregnant woman with *P. falciparum *- *P. malariae *co-infection. All previously pregnant subjects were lactating at the time of postpartum evaluation. Only two postpartum subjects were slide positive for parasitaemia, including one subject with a mixed infection.

**Table 1:** Summary of subject data for pregnant, postpartum, and non-pregnant women

	Pregnancy	Postpartum	Controls
Age (years)	23 (19 - 35)	24 (20 - 36)	24 (18 - 38)

Weight (kg)	63 (40 - 71)	55 (39 - 67)	52 (42 - 84)

BMI (kg/m^2^)	23 (17 - 27)	22 (17 - 25)	21 (17 - 28)

Parasite density at enrollment†	528 (372 - 842)	NA^‡^	807 (325 - 2215)

Baseline ALT (Units/L)	15 (8 - 31)	21 (12 - 71)	18 (11 - 67)

Baseline AST (Units/L)	26 (19 - 43)	29 (17 - 64)	32 (19 - 46)

Baseline Albumin (g/dL)	2.6 (2.1 - 3.4)	3.3 (1.8 - 5.7)	3.3 (2.8 - 4.0)

Baseline AGP (mg/dL)	70 (43 - 123)	80 (32 - 162)	99 (62 - 177)

All values given as Median (Range) unless otherwise specified.

†Values given as median (interquartile range).

^‡^Only two women in the postpartum group were slide positive for malaria.

Of the collected samples for pregnancy and control group patients, 41% of AS (40% pregnancy, 41% control) and 2% of DHA observations (<1% pregnancy, 2% control) fell below LLQ and were excluded prior to model building. One AS observation and one DHA observation were identified as outliers and excluded from the analysis. Modelling was conducted using 300 AS and 498 DHA concentrations.

### Model development

When AS data were modelled independently using first-order absorption, a two-compartment model did not improve model fit as compared to a one-compartment model. A two-compartment model was also not preferable to a one-compartment model for DHA data. A simultaneous parent-metabolite model with a one-compartment model for AS, a one-compartment model for DHA, and mixed zero-order, lagged first-order absorption was associated with lower bias in goodness-of-fit plots than simultaneous models with alternative AS absorption processes. Use of mixed zero-order, lagged first-order absorption was associated with a MOFV reduction of 665 as compared to use of a first-order absorption model.

**Figure 1:**
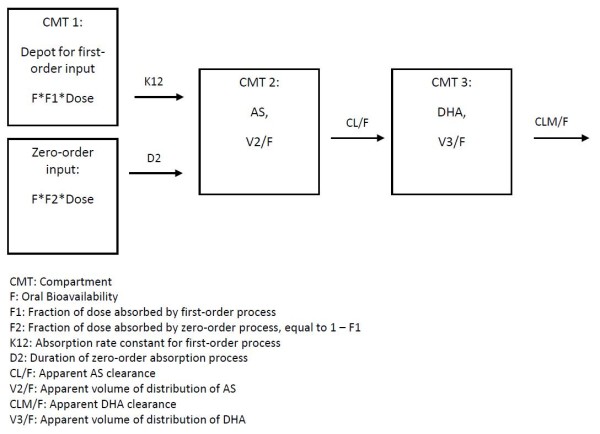
**Diagram of final structural model for AS and DHA pharmacokinetics**.

The final base model, illustrated in Figure 1, was parameterized in terms of the duration of the zero-order absorption process (D2), the rate constant of the lagged first-order absorption process (K12), the fraction of the dose absorbed by the first-order absorption process (F1), the lag time for the first-order absorption process (ALAG1), the apparent clearance of AS (CL/F), the apparent clearance of DHA (CLM/F), the apparent volume of distribution of AS (V2/F), and the apparent volume of distribution of DHA (V3/F).

The following covariate relationships were assessed during covariate modelling: pregnancy status on CLM/F, CL/F, D2, and V3/F and weight on V2/F, CLM/F, and V3/F. In the final model, only one covariate, pregnancy status on clearance of DHA, was retained as significant at the p < 0.001 level. Although not statistically significant in the full covariate analysis, the effect of pregnancy status on DHA volume of distribution was the only tested covariate relationship, apart from pregnancy status on CLM/F, that was significant (p < 0.05) in the first forward addition step of covariate modelling. Pregnancy was associated with a trend towards increased DHA volume of distribution of approximately 37%.

**Figure 2:**
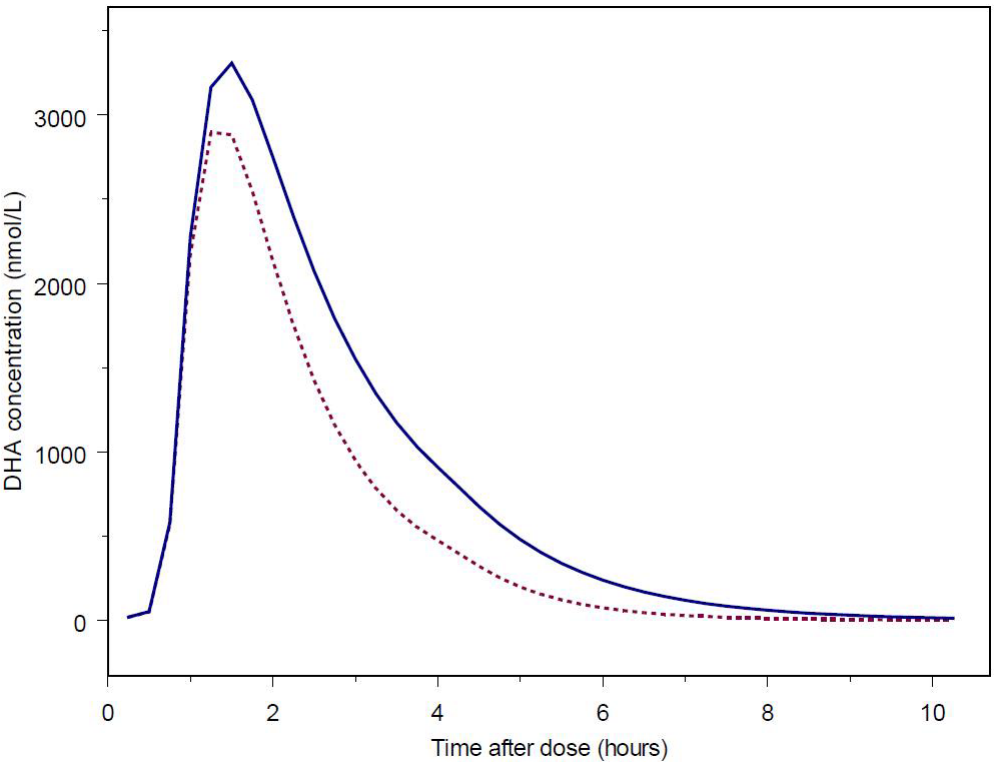
**Typical DHA concentration-time profiles for pregnant and non-pregnant women based on model parameter estimates**. The dashed line represents the typical profile for pregnant women. The solid line represents the typical profile for non-pregnant women.

The IIV values associated with CL/F and F1 were fixed after conclusion of covariate model building due to poor precision in omega estimates. NONMEM parameter estimates and relative standard errors are given in Table 2. The final parameter estimates for the model were used to generate the typical DHA concentration-time profiles, plotted in Figure 2, for the pregnant and non-pregnant women.

**Table 2:** Parameter estimates, standard error, and bootstrap confidence intervals for final model

Parameter	Estimate	%RSE	Bootstrap mean (95% CI)
K12 (h^-1^)	4.28	23.6	4.43 (2.73-7.16)

D2 (h)	4.04	19.5	3.99 (2.37-6.27)

ALAG1 (h)	0.627	10.9	0.630 (0.494-0.771)

F1	0.864	1.56	0.867 (0.839-0.887)

CL/F (L/h)	895	5.9	904 (788-1045)

V2/F (L)	195	16.4	201 (139-285)

CLM/F (L/h)	64.0	6.53	64.2 (55.1-75.2)

V3/F (L)	91.4	6.15	92.1 (78.5-109)

PREG on CLM/F	0.423	30.3	0.427 (0.197-0.723)

**IIV - Variances (%CV)**			

IIV - K12	1.84 (136)	25.3	1.76 (0.986-2.77)

IIV - D2	1.33 (115)	22.9	1.33 (0.64-2.15)

IIV - ALAG	0.573 (75.7)	20.8	0.581 (0.333-0.91)

IIV - V2/F	0.604 (77.7)	30.1	0.568 (0.253-0.942)

IIV - CLM/F	0.0802 (28.3)	24.9	0.0711 (0.031-0.113)

IIV - V3/F	0.0790 (28.1)	34.7	0.00661 (0.00591-0.139)

**RV - Variances**			

AS	0.696	11.6	0.721 (0.515-0.971)

DHA	0.174	9.94	0.174 (0.129-0.226)

RSE: Relative standard error; K12: rate of first-order absorption process for AS; D2: duration of zero-order absorption process for AS; ALAG1: lag time for first-order absorption process; F1: fraction of dose absorbed by the first-order process; CL/F: apparent clearance of AS; V2/F: apparent volume of distribution of AS; CLM/F: apparent clearance of DHA; V3/F: apparent volume of distribution of DHA; PREG on CLM/F: factor of proportional increase in CLM/F associated with pregnancy; IIV: Interindividual variability.

For postpartum data, the following absorption models, with a one-compartment model for AS and a one-compartment model for DHA, were assessed: first-order absorption, zero-order absorption, zero-order absorption with lagged first-order absorption, first-order absorption with lagged zero-order absorption, parallel dual first order absorption, single Weibull absorption, transit compartment absorption, and absorption of both AS and DHA from the gut. None of the structural models assessed provided adequate predictive power for DHA observations. Specifically, more than 20% of the DHA concentrations fell outside the visual predictive check 90% prediction interval. Attempts to model only DHA observations using various absorption processes were similarly unsuccessful.

### Model evaluation

**Figure 3:**
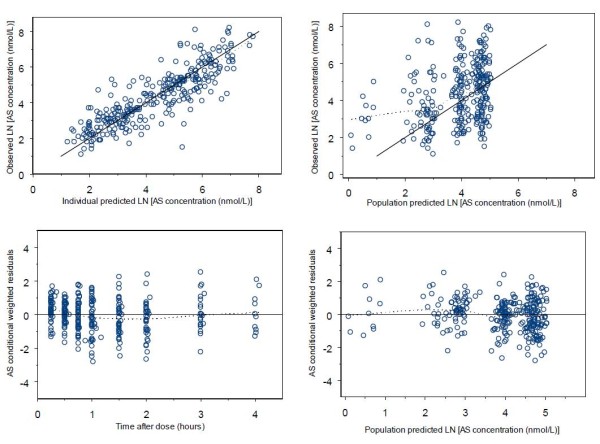
**Goodness-of-fit plots for AS**. Dotted lines are smoothing lines. Solid lines are lines of identity.

**Figure 4:**
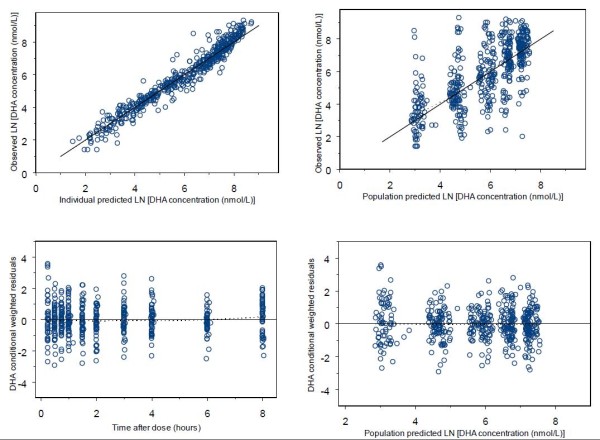
**Goodness-of-fit plots for DHA**. Dotted lines are smoothing lines. Solid lines are lines of identity.

**Figure 5:**
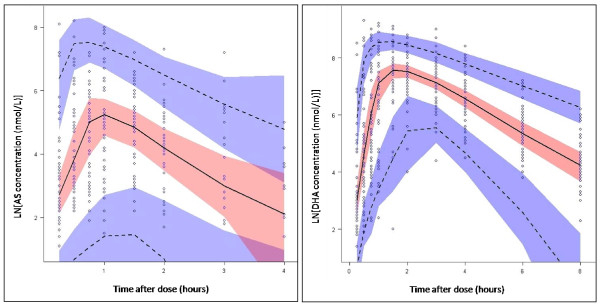
**Plot of visual predictive checks for AS and DHA**. Open circles represent observed AS and DHA concentrations. The solid line represents the median of the simulated concentrations. The dashed lines represent the 5^th ^and 95^th ^percentiles. The shaded areas represent the 95% confidence intervals for the prediction lines.

Goodness-of-fit plots for AS and DHA are given in Figures 3 and 4, respectively. Mean parameter estimates and percentile-based bootstrap 95% confidence intervals obtained from 1000 bootstrap runs are given in Table 2. Minimization was successful in 99.9% of bootstrap runs. All parameter estimates from the final model fall within the bootstrap confidence intervals. The condition number for the final model is 7.2, indicating good model stability. For the visual predictive check, 7.2% of AS and 11.2% of DHA observations fall outside the respective AS and DHA 90% prediction intervals, suggesting that the model has adequate predictive power. The visual predictive check plots for AS and DHA are in Figure 5.

## Discussion

In the present analysis, a population pharmacokinetic model was developed for AS and its active metabolite DHA using extensive sampling data from 26 pregnant and 25 non-pregnant women in the DRC. The model consists of a one-compartment model for AS and a one-compartment model for DHA, with AS absorption occurring through a mixed zero-order, lagged first-order absorption process. Upon absorption, AS is rapidly converted to DHA, with an approximate AS elimination half-life of 9.1 minutes. The model indicates that DHA apparent clearance is approximately 42% higher in pregnant than non-pregnant subjects, with resultant DHA elimination half-life estimates of 45 minutes and 59 minutes for pregnant and non-pregnant subjects, respectively.

The rapid elimination of AS found in this analysis is consistent with findings of pharmacokinetic analyses with IV AS, with an AS half-life estimate of 13.2 minutes obtained by Newton *et al *[15] and estimates of less than ten minutes found by Binh *et al *[16] and Batty *et al *[17]. Given this rapid conversion of AS to DHA, the rate of DHA formation may be limited by the rate of AS absorption. The multiple samples collected in this study during the early period following AS administration allowed for AS absorption to be characterized using a mixed zero-order, lagged first-order absorption process that offered marked improvement in model fit over simpler absorption models. Given that AS is a weak acid with a pKa of 4.6 [18], absorption though this mixed-order process may reflect AS solubility and permeability changes occurring in the differing pH environments encountered in gastrointestinal transit. Gastric absorption of AS may be limited by the solubility of the free acid form of AS (168.2 μg/mL) [19]; such solubility-limited absorption would plausibly correspond to a zero-order process [20] such as the process characterizing the initial phase of AS absorption in the mixed-order absorption model utilized in the present analysis.

Erratic AS absorption in the postpartum women appears to have contributed substantially to the difficulty in identifying a satisfactorily predictive structural model for describing data from the postpartum subjects. Given the rapid conversion of AS to DHA upon AS absorption, unpredictable AS absorption would be expected to produce a pattern of DHA appearance inconsistent with standard compartmental modelling. The source of this atypical absorption may relate to breastfeeding; the women in the study were encouraged to bring their infants to the study site and to feed the children prior to AS administration. The effects of lactation on maternal kinetics have not been extensively studied. However, some studies have been performed evaluating the effects of lactation on ethanol kinetics. These studies report changes in ethanol pharmacokinetics, which may represent altered patterns of ethanol absorption, associated with the lactational state in general, as well as more acute effects induced by breast pumping or, presumably, infant suckling [21-23]. Suckling appears to trigger the release of various hormones responsible for regulation of digestion; these hormones can alter rates of processes such as gastric emptying [21]. Therefore, it is plausible that the erratic AS absorption patterns observed for the postpartum subjects in this study may have resulted from the effects of lactation and recent infant suckling on AS absorption.

The only significant covariate identified in the present analysis was the effect of pregnancy on the clearance of DHA; this effect was estimated to produce a 42.3% (95% CI: 19.7% - 72.3%) proportional increase in DHA clearance in pregnant women as compared to non-pregnant controls. Clearance did not appear to differ substantially between the two windows of pregnancy, but more subjects in each trimester would likely be required for a difference between trimesters to be reliably detected. Apparent DHA clearance values for pregnancy and control subjects in the present analysis are similar to those obtained by non-compartmental methods, although apparent DHA volume of distribution was somewhat lower in the population, as compared to the non-compartmental, analysis [7]. In the present analysis, the apparent volume of distribution of DHA trended higher for pregnant subjects, but the association between pregnancy status and increased volume of distribution did not meet the statistical significance criteria (p < 0.001) for the described covariate analysis methods. However, given that this association was statistically significant in the initial step of covariate modelling, the association would likely attain significance if assessed in a larger number of subjects.

The source of the pregnancy-related accelerated DHA clearance identified in this analysis is difficult to determine, as pharmacokinetic changes resulting from the physiological changes of pregnancy are not presently well understood. As DHA is metabolized through hepatic glucuronidation by UGT1A9 and UGT2B7 [12], induction of these enzymes could result in accelerated DHA clearance. Induction of hepatic glucuronidation, potentially by elevated sex hormone levels in pregnancy, may be responsible for the substantial pregnancy-related increases in glucuronidation observed for various drugs, including lamotrigine [24,25], oxcarbazepine [26], and lorazepam [27]. Alterations in hepatic blood flow could also produce changes in DHA clearance. Although such alterations in blood flow during pregnancy have been investigated, the results of these investigations are not in agreement [28]. Additionally, blood flow changes may not be consistent across trimesters [29]. Therefore, the manner in which hepatic blood flow alterations would be expected to contribute to pregnancy-associated DHA pharmacokinetic changes is difficult to predict.

The results of the present analysis are comparable to those of McGready *et al*. They assessed DHA pharmacokinetics following oral AS administration to 24 pregnant women (2^nd ^or 3^rd ^trimester) of the Karen ethnic group in Thailand with acute uncomplicated falciparum malaria [5]. In their study, patients received a three-day regimen of orally administered AS (4 mg/kg/day) with 250 mg atovaquone and 100 mg proguanil; medications were given once daily with high fat milk. Samples used for pharmacokinetic analysis were obtained prior to the third daily dose and at seven time points between 0.5 and 12 hours following that dose. Since AS was detectable in only 21 of 323 samples, the investigators limited their analysis to DHA. Using population pharmacokinetic analysis, they modelled DHA data using a one-compartment model with a first-order rate of formation modelled as a fixed effect. The parameter estimates they obtained, adjusted for the median weight of their subjects (50 kg), were 88.5 L/hr [95% CI 60 - 117 L/h] for oral DHA clearance and 231.5 L [95% CI 57 - 406 L] for DHA volume of distribution. Their estimate for DHA clearance in pregnant patients is similar to the estimate in the present analysis (91 L/h). The volume of distribution estimate from the present analysis (91.4 L) is lower than found by these investigators. However, both the 91.4 L estimate and the 95% bootstrap confidence interval for that estimate (78.5-109 L), fall within their 95% confidence interval. Additionally, the 91.4 L estimate is similar to that obtained by other analysts, albeit obtained from the study of exclusively non-pregnant patients. Specifically, Teja-Isavadharm *et al *conducted non-compartmental analysis of DHA kinetics following oral AS administration to patients with falciparum malaria; their estimate of 1.33 L/kg [range: 0.70 - 2.70 L/kg] [30] is similar to the estimate obtained in the current analysis, adjusted for the median weight of the pregnancy and control groups, of 1.58 L/kg. A similar estimate of 1.33 L/kg [95% CI 1.02 - 1.64] was obtained by Newton *et al *when examining the kinetics of DHA following oral AS administration to acute falciparum malaria patients [31].

The pregnant women included in the present study were asymptomatic, displayed low-grade parasitaemia, and were otherwise generally healthy. Therefore, the results from this study can be generalized to populations for which intermittent preventative treatment regimens are indicated. Given that the model was not constructed using data from pregnant women with acute symptomatic malaria, it is not known if the model would optimally describe AS and DHA pharmacokinetics in such patients. However, given the findings of McGready *et al*, it seems probable that the significant pregnancy-associated increase in DHA oral clearance identified in the present analysis would be observed in pregnant women with acute malaria. In these patients, lower DHA blood levels resulting from accelerated DHA clearance could translate into reduced efficacy of AS and related compounds. Lower levels could also select for survival of parasites more tolerant to these compounds, increasing the risk of resistance development.

## Conclusions

In summary, this analysis describes a stable, predictive population pharmacokinetic model for AS and DHA in pregnant and non-pregnant women in the DRC. A central finding of this analysis is that an increase in DHA oral clearance is associated with pregnancy. The previous non-compartmental analysis of the data modelled in the present study found a similar difference between pregnant and non-pregnant women, but no statistically significant difference between pregnant women and the same women postpartum. However, given that the postpartum data were highly variable, the results presented here provide further support for the possibility that pregnant patients would need to receive a higher dose of AS in order to achieve equivalent DHA blood levels as obtained by non-pregnant patients receiving the standard adult dose. Although a larger study would be required to definitively characterize the optimal AS dose adjustments for pregnant patients, the substantial pregnancy-associated increase in DHA clearance described in the present analysis underscores the need for such a study.

## Competing interests

The authors declare that they have no competing interests.

## Authors' contributions

JHF, MK, EC, RWR, RSM, SRM, MD, MO, and JA made substantial contributions to the concept and design of the study; VL, JA, MO, DW, SRM and LF were involved in data acquisition; MK, EC, LF, CAM, CB, SM, DW, AKT, SRM and MO contributed to data analysis and interpretation. All the authors critically reviewed the paper and approved the final version for submission.
